# Two-Dimensional Semiconductor Optoelectronics Based on van der Waals Heterostructures

**DOI:** 10.3390/nano6110193

**Published:** 2016-10-27

**Authors:** Jae Yoon Lee, Jun-Hwan Shin, Gwan-Hyoung Lee, Chul-Ho Lee

**Affiliations:** 1 KU-KIST Graduate School of Converging Science and Technology, Korea University, Seoul 02841, Korea; jylee89@korea.ac.kr (J.Y.L.); flyeye18@gmail.com (J.-H.S.); 2 Department of Materials Science and Engineering, Yonsei University, Seoul 03722, Korea

**Keywords:** 2D semiconductors, transition metal dichalcogenides, van der Waals heterostructures, optoelectronics, photodetectors, solar cells, light-emitting diodes

## Abstract

Two-dimensional (2D) semiconductors such as transition metal dichalcogenides (TMDCs) and black phosphorous have drawn tremendous attention as an emerging optical material due to their unique and remarkable optical properties. In addition, the ability to create the atomically-controlled van der Waals (vdW) heterostructures enables realizing novel optoelectronic devices that are distinct from conventional bulk counterparts. In this short review, we first present the atomic and electronic structures of 2D semiconducting TMDCs and their exceptional optical properties, and further discuss the fabrication and distinctive features of vdW heterostructures assembled from different kinds of 2D materials with various physical properties. We then focus on reviewing the recent progress on the fabrication of 2D semiconductor optoelectronic devices based on vdW heterostructures including photodetectors, solar cells, and light-emitting devices. Finally, we highlight the perspectives and challenges of optoelectronics based on 2D semiconductor heterostructures.

## 1. Introduction

Inspired by the great success of graphene, the emerging research on a new class of atomically thin two-dimensional (2D) materials with diverse physical properties has become compelling in recent years [1,2,3,4,5,6,7,8,9,10,11,12,13,14,15,16,17]. Although graphene has remarkable electronic, thermal and mechanical properties [18,19,20,21,22], due to its zero bandgap, it possesses an inherent limitation for applications in various electronic and optoelectronic devices. Therefore, very recently, many researchers have paid significant attention to other 2D materials that have finite bandgap energies [23,24,25].

**Figure 1:**
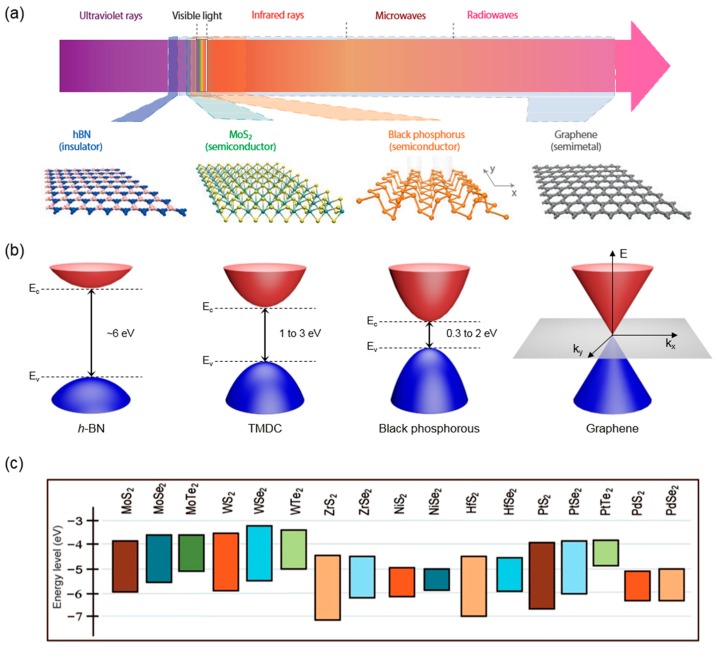
(**a**) Energy spectrum of various two-dimensional (2D) materials and their atomic crystal structures [27]; (**b**) electronic band structures of hexagonal boron nitride (*h*-BN), transition metal dichalcogenides (TMDCs), black phosphorous, and grapheme; and (**c**) energy level diagrams of the selected semiconducting TMDCs [6]. Reproduced with permission from [6,27]. Copyright Nature, 2014 and Copyright Wiley, 2015, respectively.

In fact, in the realm of nature, there are a large number of 2D materials including the graphene family, metal chalcogenides and metal oxides [5,6,26,27,28]. These materials can cover a broad range of electrical and optical properties, as shown in Figure 1a,b [27]. For the 2D semiconductors, first of all, a variety of transition metal dichalcogenides (TMDCs) including group IV (Mo and W) dichalcogenides are available. Since semiconducting TMDCs have sizable bandgap, unlike graphene, they have been exploited for electronic devices including field-effect transistors (FETs) and memories [29,30,31,32]. They are also promising optical materials whose energy spectrum is ranged from visible to near infrared wavelength [13,14,15]. More interestingly, these atomically thin 2D semiconductors have exceptional optical properties including the indirect-to-direct bandgap transition at the monolayer regime [23,33], rich spin-valley interplays [34,35,36], strong light–matter interactions [37,38,39], and large exciton bind energy [33,40,41,42,43], which will be further discussed in the following section. As well as semiconducting TMDCs, very recently, black phosphorous has emerged as a new 2D semiconductor [25,44]. Distinctively, it has a tunable direct bandgap, largely varying from 0.3 eV to ~2.0 eV with reducing the thickness [45,46]. For the large bandgap insulators, a hexagonal boron nitride (*h*-BN) with the bandgap of ~6 eV is the most famous these days [3]. Because the *h*-BN has atomically flat and smooth surface with lack of dangling bonds, it has been successfully used as an ideal substrate for other 2D conducting channels such as graphene and TMDCs [47,48,49]. In addition to *h*-BN, 2D metal oxide nanosheets with various compositions have been investigated as a high-*k* dielectric layer. Lastly, for the 2D metals, numerous metallic TMDCs including group V compounds are also available as well as graphene [4]. Table 1 summarizes currently available 2D materials that are electrically metallic, semiconducting and insulating.

The availability of 2D materials with various compositions and electronic structures enables one to stack these layers with atomic precision and to produce artificial van der Waals (vdW) heterostructures that are essentially different from conventional material heterostructures as further discussed in the Section 3 [50,51,52,53]. Such ability offers an exciting opportunity in new materials design for fundamental studies and device applications [7,8,9,10]. In particular, as shown in Figure 1c, because semiconducting TMDCs have different band gap energies and work functions, it is possible to build atomically thin semiconductor heterojunctions and/or superlattices with designed band alignment [26,27,28]. That enables investigating the unexplored optical and optoelectronic properties in an ultimate 2D limit, and also leads to novel device concepts and technologies for the next-generation optoelectronics. In this article, we mainly review the unique characteristics of 2D semiconductors including the electronic band structures and optical properties and highlight the recent progresses on the fabrication of novel optoelectronic devices based on 2D semiconductor vdW heterostructures.

## 2. Transition Metal Dichalcogenides as 2D Semiconductors

**Figure 2:**
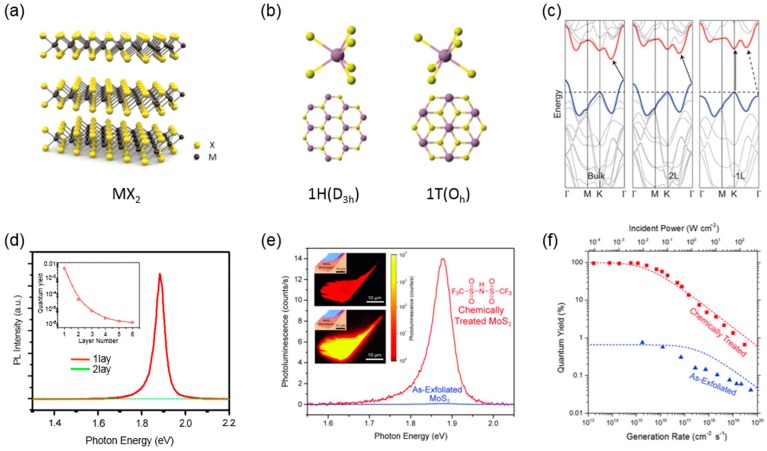
(**a**) Crystal structure of a transition metal dichalcogenide (TMDC), MX_2_ [29]; (**b**) atomic arrangement of two structural polymorphs of MoS_2_ identified as one hexagonal (1H), one tetragonal (1T) [54]; (**c**) band structures of bulk, bilayer (2L) and monolayer (1L) MoS_2_ [56]; (**d**) Photoluminescence (PL) spectra for 1L and 2L MoS_2_, Inset: quantum yield as a function of the number of layers [23]; (**e**) PL spectra for as-exfoliated and TFSI-treated MoS_2_ monolayers, Inset: PL intensity maps for each samples; and (**f**) power dependence of the quantum yield for as-exfoliated and chemically-treated MoS_2_ [62]. Reproduced with permission from [29]. Copyright Nature, 2011, [54] Copyright Nature, 2014, [56] Copyright American Chemical Society, 2010, [23] Copyright American Physical Society and [62] Copyright Science, 2015, respectively.

Among numerous layered materials, TMDCs as an atomically thin 2D semiconductor have recently attracted considerable interest for potential applications in diverse electronic and optoelectronic devices [7,8,9,10,11,12,13,14,15,16]. TMDCs are compound semiconductors with composition of MX_2_, where M is the transition metal atoms from groups IV to VII elements in the periodic table and X is the chalcogen atoms such as S, Se and Te [4]. Unlike the one-atom-thick graphene, as shown in Figure 2a, a TMDC is three-atom-thick, in which the covalently-bonded atomic plane of transition metals is sandwiched between two atomic planes of chalcogen atoms [29]. Each monolayer is held together by weak vdW forces in the out-of-plane direction, forming a layered structure. As mentioned above, a variety of TMDCs are available, whose electrical properties are either metallic or semiconducting depending on the constituent transition metal atoms [4]. Even among semiconducting TMDCs, the electromagnetic spectrum is widely ranged from visible to infrared wavelength range [27].

Semiconducting TMDCs have a variety of polymorphs depending on atomic arrangement of chalcogen atoms. For example, MoS_2_, one of the most famous group VI TMDCs, has two polymorphs of 1T and 2H phases (Figure 2b) [4]. 1T-MoS_2_ has tetragonal symmetry with octahedral (O_h_) coordination, whereas 2H-MoS_2_ has a hexagonal crystal structure with trigonal prismatic (D_3h_) coordination. Due to this structural difference, they have completely different electronic band structures: 1T-MoS_2_ is metallic and 2H-MoS_2_ is semiconducting with sizable bandgap. Nevertheless, a 2H semiconducting TMDC has been widely studied since it is more thermodynamically stable than a 1T metallic phase. Recently, Kappera et al. reported the 1T metallic phase formed by local phase transition only at the contact area can be utilized to reduce the contact resistance of FETs with the 2H MoS_2_ channel [54].

As well as the atomic structure, semiconducting TMDCs have distinctive and remarkable optical properties compared with conventional bulk semiconductors. It is well known that TMDCs exhibit the unusual transition of the electronic band structure from indirect to direct bandgap when thinning down to the monolayer [23,33]. In the electronic band structure shown in Figure 2c, since the states at Γ-point have a strong *p*-orbital character of anion X, they shift upward due to interlayer orbital interaction in the case of multilayer TMDCs. Whereas, the states at *K*-point that have a *d*-orbital character of cation M are dominantly localized in the X-M-X structure and marginally affected by the number of layers [4,33]. These detailed origins of electronic band structures lead to evolution of an indirect bandgap transition when the TMDC become thicker than bilayer [55].

Because of such an extraordinary change of the band structure, the photoluminescence (PL) characteristics of monolayer TMDCs can be drastically enhanced [23,56]. In spite of much increased PL intensity, the absolute quantum efficiency was still quite low due to non-radiative recombination processes mainly mediated by defect sites such as vacancies and impurities [56,57,58,59,60,61]. In case of the monolayer MoS_2_ exfoliated from single crystals, the PL quantum yield was reported in the range of 0.01%–0.6% [23,58,59]. To overcome such an issue, there have been many attempts including thermal annealing and chemical treatments [60,61], which can suppress the defect–mediated and/or Auger non-radiative recombination processes. As shown in Figure 2e,f, Amani et al. at University of California at Berkley have recently demonstrated that the PL quantum yield can be drastically increased near unity (~95%) by simply dipping the monolayer MoS_2_ into an organic-based superacid (Bis(trifluoromethane)sulfonimide, TFSI) solution [62,63]. They suggested that the organic acid can compensate defect sites such as adatoms and impurities and can passivate sulfur vacancies, leading to significant enhancement of PL quantum yield and minority carrier lifetime by more than two orders of magnitudes. These results offer an important breakthrough in utilizing TMDCs as an optically active material for many optoelectronic devices, particularly for light-emitting devices.

Semiconducting TMDCs have strong light–matter interaction due to band nesting and van Hove singularities in the density of states, resulting in considerable amount of light absorption particularly in the visible wavelength range [37,64]. Many optical spectroscopic studies have revealed that the monolayer TMDCs including MoS_2_, MoSe_2_, WS_2_, and WSe_2_ can absorb up to 5%–10% of incident visible light despite its atomic thickness (less than ~1 nm), which is about one order of magnitude higher than that of traditional semiconductors such as GaAs and Si assuming the same thickness [65,66]. Furthermore, atomically thin 2D semiconductors have extremely large exciton energy, which is orders of magnitude larger than those of conventional inorganic semiconductors due to the quantum confinement effect and reduced dielectric screening. Previous theoretical calculations predict that the exciton binding energies of monolayer group VI TMDCs is in the range of 0.3–1.0 eV [33,40,41,42,43]. Ugeda et al. experimentally determined the exciton binding energy of MoSe_2_ on graphene to be 0.55 eV by means of scanning tunneling spectroscopy and PL spectroscopy [42]. Chernikov et al. also reported that WS_2_ has the exciton binding energy of 0.32 eV [43]. Due to extreme quantum confinement, in addition, the tightly-bound trions, a three-body quasiparticle composed of an exciton (an electron–hole pair) and a charge carrier (an electron or a hole), have been observed in the monolayer TMDCs even at room temperature [67]. Such a large excitonic effect and strong light–matter interaction of atomically thin 2D semiconductors make them promising for the fabrication of high-performance ultrathin optoelectronic devices [13,14,37]. Furthermore, TMDCs can offer additional advantages for next-generation optoelectronics due to their optical transparency, mechanical flexibility and non-epitaxial fabrication on arbitrary substrates [13,68].

## 3. Van der Waals Heterostructures Built from Various 2D Materials

### 3.1. Features of 2D van der Waals Heterostructures

**Figure 3:**
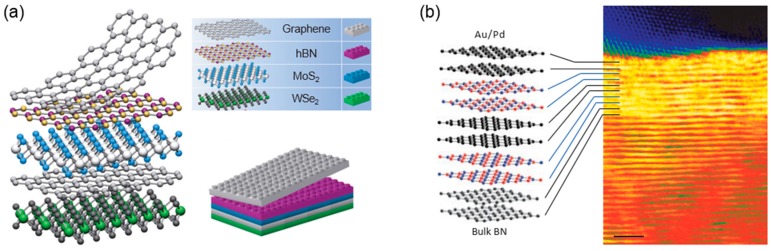
(**a**) Schematic diagram of 2D vdW heterostructures composed of graphene, *h*-BN, and TMDCs [51]; and (**b**) cross-sectional scanning transmission electron microscopy (STEM) image of a graphene/*h*-BN heterostructure. Reproduced with permission from [51,70]. Copyright Nature, 2012.

The common feature of 2D layered materials is that the covalently-bonded atomic layers are held together by weak vdW interaction. Because of that, a large number of 2D atomic crystals could be mechanically exfoliated from bulk single crystals. More interestingly, because there are no physical bonds between the layers, it is possible to mechanically stack arbitrary 2D materials together without atomically precise commensurability just as assembling the atomic-scale *lego* blocks (Figure 3a) [51]. This offers a lot of merits and degrees of freedom in fabricating the heterostructures based on 2D materials [69]. In principle, a variety of different layered constituents enable one to build functional 2D heterostructures with countless combinations, whose heterointerfaces can offer completely different types of 2D electronic systems. Furthermore, 2D vdW heterostructures are essentially distinct from conventional covalently- or ionically-bonded three-dimensional (3D) materials in the way that a vdW surface with free of dangling bonds allows us to create high-quality interfaces [51,52]. As shown in scanning transmission electron microscopy (STEM) images of Figure 3b, the atomically abrupt and chemically clean interfaces can be easily achieved between different 2D materials [70]. Generally, in conventional 3D bulk materials, the fabrication of high-quality heterostructures is strictly limited by material mismatches in crystal structures, lattice constants and thermal expansion coefficients. In addition, it requires the advanced growth techniques such as molecular beam epitaxy and metal-organic chemical vapor deposition (CVD).

The capability to build atomically controlled heterostructures enables to investigate the intrinsic materials properties and unexplored physical phenomena in an ultimate 2D limit. For example, high mobility approaching to theoretical values has been reported from both graphene and MoS_2_ sitting on the *h*-BN dielectric layer [47,48,49]. Exotic physical phenomena such as fractional quantum Hall effect, resonant tunneling and Coulomb drag have been explored in the graphene/*h*-BN heterostructures [71,72,73,74,75]. As well as fundamental studies, the novel device concepts based on 2D vdW heterostructures have been proposed for a variety of electronic and optoelectronic device applications including field-effect tunneling transistors [76,77], charge trapping memories [31,32], ultrafast photodetectors [78,79,80], and 2D light-emitting didoes (LEDs) [81,82,83].

In addition to experimental efforts, it is very important to develop accurate and effective theoretical approaches for modeling the electronic band structures and physical properties of 2D vdW heterostructures [26,84,85]. Therefore, many researchers have studied the electronic structure and optical properties of a variety of 2D vdW heterostructures using first principles calculations although there are certain limitations such as huge amount of calculation and incongruity of incommensurate interfaces [33,86,87,88]. For example, the interlayer interaction and band structures in commensurate bilayer TMDC heterojunctions have been investigated [41,89]. In addition, Bernardi et al. computed the light absorption of individual TMDC monolayers and predicted the performance of TMDC heterojunction solar cells using the combined density functional theory and GW-Bethe Salpeter equations [65]. Recently, using a multiscale approach, Andersen et al. have successfully calculated the dielectric properties of incommensurate vdW heterostructures comprising hundreds of layers [90].

### 3.2. Fabrication of 2D van der Waals Heterostructures

**Figure 4:**
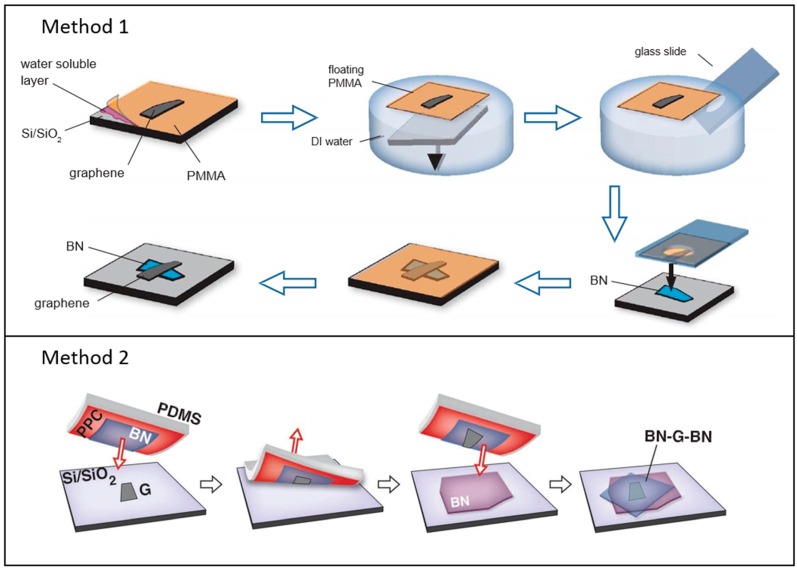
Representative methods for fabricating 2D vdW heterostructures (i.e., graphene/*h*-BN stacks). Method 1 is a technique based on the mechanical transfer and staking using a polymer supporting layer [50]. Method 2 is an assembly technique based on the mechanical transfer and pick-up processes [91]. Reproduced with permission from [50]. Copyright Elsevier, 2012 and [91] Copyright Science, 2013, respectively.

For the vdW assembly of numerous 2D materials in the out-of-plane direction, there are mainly two skillful techniques based on direct mechanical exfoliation, transfer, and stacking methods, enabling the fabrication of a variety of vdW heterostructures. Figure 4 shows the schematic illustrations of two representative methods developed by researchers at Columbia University [50,91]. One method is to put one of materials onto the other 2D materials or substrates by using an elastomer stamp combined with an adhesive polymer supporting layer [50]. As shown in Figure 4a, one of 2D crystals is exfoliated onto the polymer layers consisting of the supporting polymethylmethacrylate (PMMA) layer and the water-soluble sacrificial layer coated on the SiO_2_/Si substrate. Then, by dissolving water-soluble layer, the PMMA layer with the exfoliated flake is floated on the de-ionized water. The floated PMMA layer is lifted up with the polydimethylsiloxane (PDMS) stamp attached on the transparent glass. After precise alignment using a micromanipulator, the 2D crystal is transferred onto the other crystal separately exfoliated on the SiO_2_/Si substrate by melting down the PMMA layer on a heating stage. After that, PMMA is removed by soaking in acetone. These processes can be applied to both the mechanically-exfoliated samples from bulk single crystals and CVD-grown 2D materials. Although this method is quite useful and successful for fabricating the heterojunction between the two layers, it has an inherent drawback for creating the multilayer heterostructures due to the imperfect cleanliness of residual polymers at the interfaces. That limits to realize the various combination of multilayer stacking while maintaining the atomically sharp interfaces. The second method is developed to avoid interface contamination by polymer residues during transfer processes [91]. A 2D crystal that was put on an adhesive polymer stamp is used to pick up another 2D material through vdW attraction acting on each other. As shown in Figure 4b, the atomically flat 2D material such as *h*-BN placed on the polypropylene carbonate (PPC)/PDMS structure is brought into contact with the graphene layer, then the stamp layer (*h*-BN) picks up the target layer (graphene) from the SiO_2_/Si substrate. By repeating this process several times, one can make multiple-layered assemblies with precisely controlled sequences and atomically sharp interfaces among the neighboring layers. 

The above-mentioned methods based on mechanical exfoliation, transfer and stacking have been widely used for fundamental studies to investigate the intrinsic materials properties and exotic physical phenomena in a variety of novel 2D vdW heterostructures. However, these stacking methods have critical drawbacks for practical applications including difficulty in large-area scaling, time-consuming processes and low production yield. Using the large-scale CVD methods, thus, the direct growth methods of vertical (out-of-plane) and lateral (in-plane) heterostructures have recently been demonstrated [92,93,94,95,96]. Especially, epitaxially-grown vertical heterostructures, in contrast to mechanically-stacked heterostructures with the randomly-twisted angles, have the specific relationship of crystallographic orientations between the constituent monolayers [38,97]. Although CVD-grown 2D materials can also be utilized to compose the mechanically-stacked vdW heterostructures, for large-area optoelectronic applications, it is still required to develop reliable and scalable growth techniques for preparing high-quality 2D materials and their heterostructures with precisely controlled compositions, atomically clean and sharp interfaces.

## 4. Optoelectronic Devices Based on 2D Semiconductor Heterostructures

As discussed above, a large number of 2D semiconductors with variable bandgap and work function are available in the realm of nature. They also have unique and excellent optical properties including the indirect-to-direct bandgap transition at the monolayer regime, strong light–matter interaction and large exciton binding energy. Combining with the ability to create the artificial 2D vdW heterostructures, it is possible to design the novel 2D optoelectronic devices such as photodetectors, solar cells, LEDs and laser [37,80,81,82,83,98,99,100,101,102,103]. As earlier works, the lateral *p*-*n* junctions have been reported by several research groups through spatially-split electrostatic gating in a single TMDC channel, demonstrating the optoelectronic functions such as solar-energy harvesting and visible-light emitting [104,105,106,107,108,109]. However, we here focus on presenting the recent progresses on 2D semiconductor optoelectronic devices based on vertically-assembled vdW heterostructures.

### 4.1. Photodetection and Photovoltaic Devices

#### 4.1.1. Photodetection Based on Graphene/TMDC/Graphene Heterostructures

**Figure 5:**
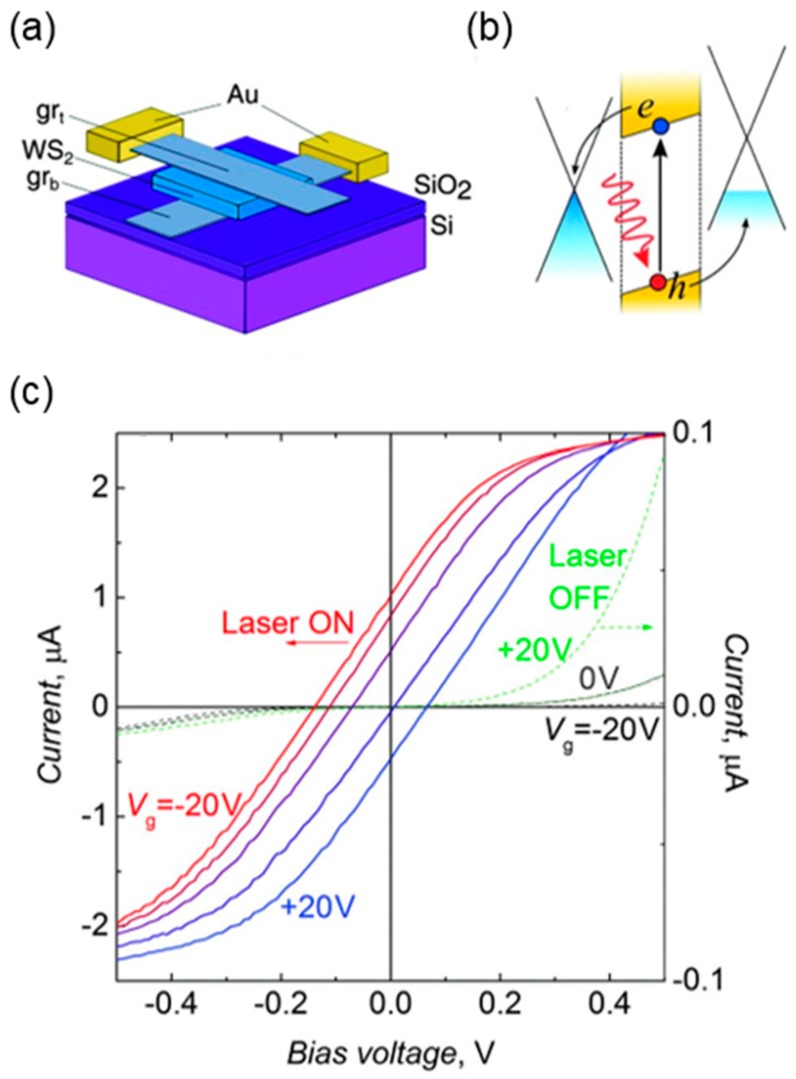
Photodetection device based 2D vdW heterostructure composed of graphene/WS_2_/graphene: (**a**) Schematic illustration; (**b**) band alignment; and (**c**) photoresponse characteristics as a function of the gate voltages. Reproduced with permission from [37]. Copyright Science, 2013.

As a pioneering work, in 2013, Britnell and colleagues at Manchester University reported the 2D photodetectors utilizing a vertical vdW heterostructure composed of semiconducting TMDCs such as WS_2_ and MoS_2_ as absorption layers sandwiched between metallic graphene layers [37]. Using the typical mechanical transfer and stacking methods, as shown in Figure 5a, they realized the photoresponsive device consisting of the graphene/TMDC/graphene stack where graphene that is optically transparent, electrically conducting and electrostatically tunable was employed as the top and bottom electrodes. Fermi levels of the two graphene electrodes can be positioned differently by electrostatic doping with the gate voltage, creating the built-in potential gradient within the TMDC layer across the top and bottom graphene electrodes (Figure 5b). That makes it possible to dissociate the electron–hole pairs excited in the TMDC layer by incident light, generating the photocurrent. In this structure, because the Fermi level of the bottom graphene is effectively modulated by electric field effect, the device showed the tunable electrical and photoresponse characteristics upon varying the gate voltage (Figure 5c). In addition, the absolute light absorption of the device can be further increased by simply using multilayer TMDCs and additionally utilizing the plasmonic effect of metal nanostructures deposited on the top surface [37,110]. The external quantum efficiency (EQE) of the device is achieved above 30% [37]. Similarly, the highly gate-tunable photoresponse characteristics in various graphene/TMDC/graphene heterostructures were reported by the researchers at University of California at Los Angeles [98].

Another strength of vertical vdW heterostructures is that it enables the high-speed photodetection at the picosecond scale and further improvement of quantum efficiency owing to extremely short transport length of charge carriers (or excitons) within atomically thin active layers. Recently, Massicotte et al. have demonstrated that the optimum photoresponse time can be as short as 5.5 picoseconds and the EQE reaches up to 7.3% from the *h*-BN-encapsulated graphene/WSe_2_/graphene heterostructure by using time-resolved photocurrent (two-pulse correlation) measurements [80]. The ultrafast photoresponse was also tunable depending on the applied gate voltage and the thickness of TMDC layers. By reducing the channel length, one could achieve the enhanced internal quantum efficiency as well as faster photoresponse time because of shortening of carrier extraction time relative to carrier recombination lifetime.

#### 4.1.2. Atomically Thin *p-n* Heterojunctions Based on Semiconducting TMDCs

As well as a single TMDC layer, the TMDC heterojunctions can be employed as optically active layers in various photoconversion devices. A *p*-*n* junction would be the simplest one that has been widely used as a fundamental unit for various semiconductor optoelectronic devices such as photodetectors, solar cells, and LEDs [81,99,100,101,102].

**Figure 6:**
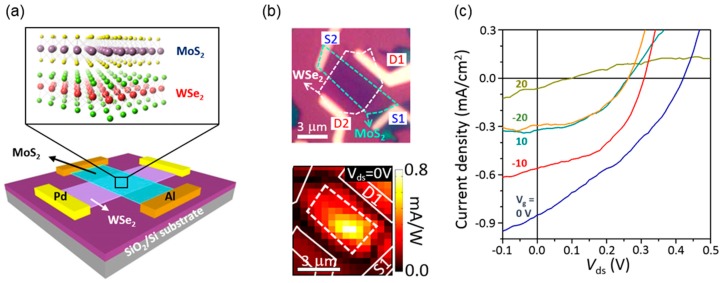
Gate-tunable photovoltaic response in an atomically thin *p*-*n* heterojunction. (**a**) Schematic diagram of a vdW-stacked MoS_2_/WSe_2_ heterojunction device with lateral metal contacts; (**b**) Optical image of the fabricated device (top) and photocurrent map of the device for *V*_ds_ = 0 V and 532 nm laser excitation (bottom). The junction area and metal electrodes are indicated by dashed and solid lines, respectively; (**c**) Photoresponse characteristics at various gate voltages under white-light illumination. Reproduced with permission from [99]. Copyright Nature, 2014.

Recently, the researchers at Columbia University reported the fabrication of atomically thin *p*-*n* heterojunctions with two different device geometries and thoroughly investigated their optical and optoelectrical characteristics [99]. As shown in Figure 6a, they fabricated the heterojunction by vertical staking of the *n*-type MoS_2_ monolayer and *p*-type WSe_2_ monolayer, resulting in a *p*-*n* heterojunction with the type-II band alignment. The device laterally contacted with metal electrodes exhibited the gate-tunable diode behaviors. In addition, the photovoltaic responses mostly originated from the heterojunction area were observed in an atomically thin *p*-*n* junction (Figure 6b,c). Although the junction seems to exhibit very similar device characteristics compared with those of conventional bulk *p*-*n* junctions, authors found that the underlying operation principle of the device is completely different from that of bulk counterparts due to absence of the extended depletion region and carrier diffusion in an atomically thin *p*-*n* junction. Despite large exciton binding energy at room temperature, the electron–hole pairs generated in each material can be separated easily by strong internal electric field formed at the type-II heterointerface, which is also confirmed by strong PL quenching resulted from charge transfer. In addition, the ultrafast charge transfer might be expected across the atomically thin heterojunction because diffusion of excitons (or minority carriers) is not required. As the separated experimental results, Hong et al. at University of California at Berkeley reported that charge transfer occurs at a very fast rate of ~50 femtoseconds in the MoS_2_/WS_2_ heterostructure with the similar type-II band alignment [78].

Even after efficient and fast charge separation, the electrons and holes can recombine each other again through interlayer recombination processes through quantum tunneling because the separated carriers are still confined spatially within adjacent 2D atomic planes. This interlayer tunneling is understood by two physical mechanisms: (1) Langevin recombination driven by Coulomb interaction; and/or (2) Shockley–Read–Hall recombination mediated by trap states present in the middle of the bandgap. The researchers at Columbia University have proved that the electrical and photoresponse characteristics in an atomically thin *p*-*n* junction are determined by interlayer tunneling recombination instead of drift and diffusion of carriers in a bulk junction. Table 2 summarizes the remarkable differences between atomically thin and bulk *p*-*n* junctions in terms of the band structure and underlying physical principle.

**Figure 7:**
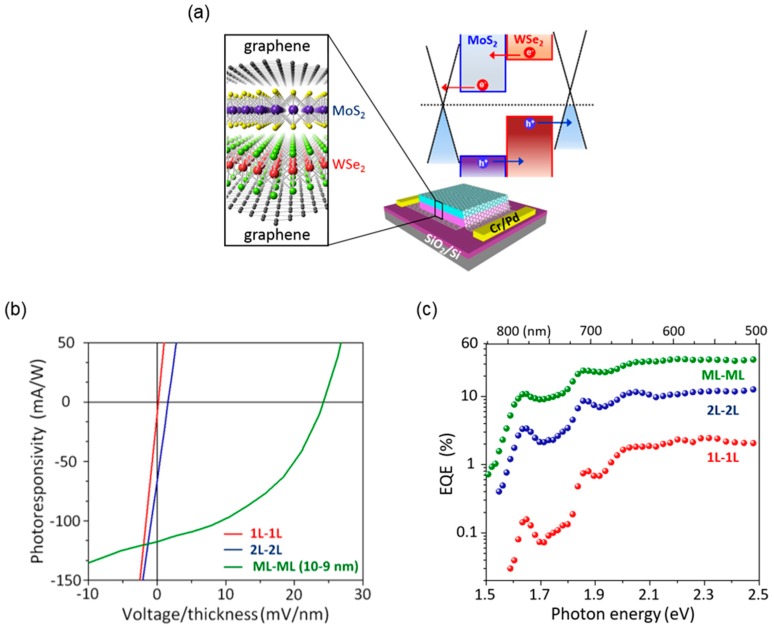
Photodetection and/or photovoltaic devices based on the 2D semiconductor heterostructure consisting of graphene/TMDC *p*-*n* junction/graphene: (**a**) schematic illustration of the atomic crystal structure, band alignment, and device geometry; (**b**) photoresponse characteristics; and (**c**) the measured external quantum efficiency (EQE) as a function of excitation wavelength for the devices with different thicknesses of the *p*-*n* junctions. Reproduced with permission from [99]. Copyright Nature, 2014.

The interlayer-tunneling-mediated recombination process that is prohibited in a bulk *p*-*n* junction decreases the quantum efficiency of the photon-to-electron conversion. To resolve such an issue, as shown in Figure 7a, they proposed the all-2D-vdW heterostructure consisting of the graphene/MoS_2_/WSe_2_/graphene stack, where graphene layers are used as electrodes for charge collection. In this geometry, as well as the dissociation of excitons at the semiconductor interface, the extraction of carriers at the graphene/TMDC interface occurs very quickly through quantum tunneling. This suppresses the recombination processes after the separation of excitons so that it can enhance the overall photoconversion efficiency. These results suggest that the vertical all-2D-vdW heterostructures can be exploited to realize the high-efficiency photovoltaic devices and ultrafast photodetectors.

Although the graphene-sandwiched *p*-*n* heterostructure can be utilized as a photodetector, it may not be suitable for applications in solar cells because it cannot sustain the photovoltage across the top and bottom graphene electrodes due to direct tunneling current between them. By simply increasing the total thickness of the *p*-*n* junction to reduce the tunneling current, the typical photovoltaic property with finite values of short-circuit current and open-circuit voltage was restored (Figure 7b). Furthermore, as shown in Figure 7c, the 20-nm-thick *p-n* junction device exhibits the EQE above 30%. These experimental results suggest that the TMDC heterojunctions with clean vdW interfaces can be useful for solar cells even if the TMDC is not monolayer. For the practical applications, however, it is required not only to optimize the power conversion efficiency by considering both light adsorption and diffusion length of excitons (or minority carriers), but also to develop the strategies for the large-scale growth and fabrication. Furthermore, the power conversion efficiency can be further improved by increasing the light adsorption using surface plasmon resonance effect or designing of the layered tandem structures using various 2D semiconductors with different band gap energies.

### 4.2. Light-Emitting Devices

2D semiconductors such as TMDCs and black phosphorous have several useful properties for light emission as well as light absorption. As previously mentioned, in particular, the monolayer TMDCs are direct bandgap semiconductors. Black phosphorous is also a direct bandgap material regardless of the thickness [45,46]. In general, it is very important to have the direct bandgap semiconductors for realizing high-efficiency light-emitting devices because the direct bandgap transition have much higher quantum efficiency than the indirect transition that requires the additional phonon energy to conserve the momentum. In addition, the large exciton binding energy of these materials can lead to enhancement of radiative recombination rate. These unique properties of 2D semiconductors may allow one to fabricate ultrathin 2D light emitters with high efficiency.

Light-emitting device based on 2D semiconductor heterostructures has been first reported in the aforementioned *p*-WSe_2_/*n*-MoS_2_ heterojunction [81]. Cheng et al. observed the electroluminescence (EL) occurred by hot electrons or recombination of electrons and holes injected in each layer depending on the applied voltages. Furthermore, the monolayer device exhibited stronger EL than the multilayer one. However, the observed EL has relatively low quantum yield because the ultrafast and efficient charge transfer rather than carrier confinement takes place in the type-II heterointerface. Besides, the type-II transition at the vertically-stacked *p*-*n* junction with randomly-twisted angles may lead to low quantum yield of radiative recombination due to the momentum mismatch between electrons (conduction band of MoS_2_) and holes (valence band of WSe_2_).

**Figure 8:**
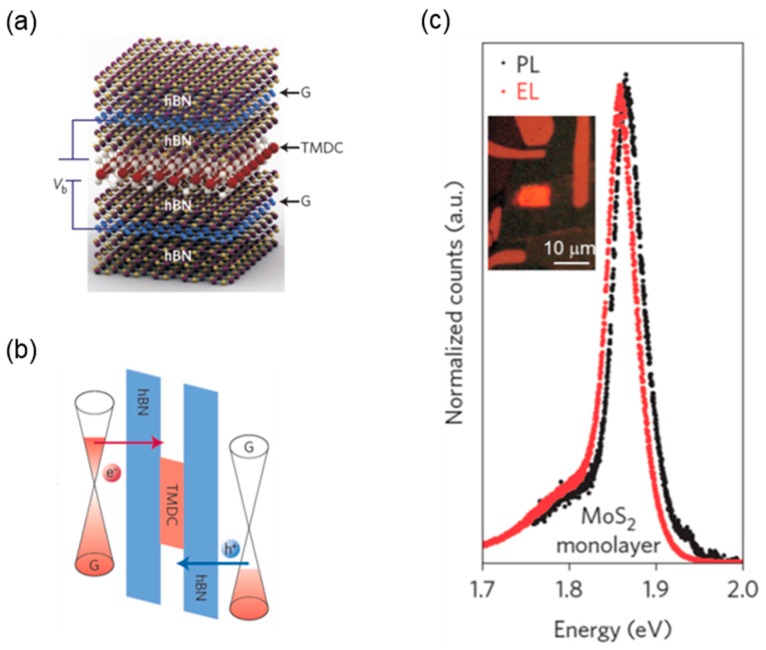
Light-emitting devices based on the 2D vdW heterostructure of graphene/*h*-BN/TMDC/*h*-BN/graphene: (**a**) schematic diagram; and (**b**) band alignment of the heterostructure; and (**c**) PL and EL spectra, and the EL image (inset) measured from the device. Reproduced with permission from [82]. Copyright Nature, 2014.

To achieve the high luminescence efficiency, the injected charge carriers should be confined effectively within the emitting layer. In case of traditional compound semiconductor LEDs, the bandgap-engineered multiple quantum wells (MQWs) have been employed in the emission region between *n*-type and *p*-type injection layers [111]. Recently, the researchers at Manchester University proposed a similar strategy with 2D vdW heterostructures by bandgap engineering [82]. As shown in Figure 8, they fabricated 2D light-emitting devices composed of graphene/*h*-BN/monolayer TMDC/*h*-BN/graphene heterostructures. In this device, the atomic-scale single quantum well was built by using the *h*-BN as a barrier and the monolayer TMDC as an emitting layer. Furthermore, the metallic graphene layers were employed as electron and hole injection layers. Then, the electrically injected electrons and holes in the monolayer TMDC sandwiched between the large bandgap *h*-BN layers can radiatively recombine well each other due to effective carrier confinement. Furthermore, by repeating the *h*-BN/TMDC/*h*-BN structure several times, they managed to fabricate the MQWs or superlattice structures. The device with MQWs exhibited the external quantum yield of ~10%, which is comparable to that of the organic LEDs [82].

**Figure 9:**
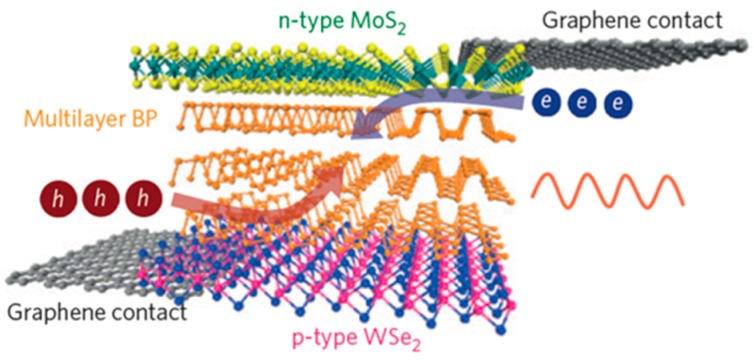
The recently proposed infrared LEDs based on 2D vdW heterostructures using black phosphorous as an emitting layer. Reproduced with permission from [27]. Copyright Nature, 2014.

In order to realize high-efficiency LEDs, the monolayer TMDCs should be used as an emitting layer. In addition, the emission wavelength is mostly limited in the visible range which corresponds to bandgap energy of TMDCs. Due to its thickness-independent direct bandgap and widely tunable bandgap of 0.3–2 eV, black phosphorous has been suggested as an alternative luminescent material for 2D LEDs particularly in the mid-infrared range. In the proposed device geometry illustrated in Figure 9, the black phosphorous layer that has a narrow bandgap is sandwiched between the electron-injecting MoS_2_ and hole-injecting WSe_2_ layers that have larger bandgap than black phosphorous, enabling both efficient injection and confinement of charge carriers [27].

These results suggest that the bandgap-engineered 2D vdW heterostructures can be applied to high-efficiency 2D planar light-emitting devices. Furthermore, in contrast to the conventional compound semiconductor LEDs based on epitaxy, it is possible to manufacture the light emitters on a variety of non-epitaxial substrates including glass, plastic, metal and silicon, which can offer significant opportunities for emerging applications in smart windows, flexible displays and optoelectronic circuits.

## 5. Conclusions and Perspectives

In this short review, we presented the latest research progresses on the fabrication of 2D semiconductor vdW heterostructures and their optoelectronic device applications including light-harvesting and light-emitting devices. Owing to the exceptional optical properties of 2D semiconductors and unique capability to create the vdW heterostructures with atomically sharp interfaces, various optoelectronic devices based on 2D semiconductor heterostructures, including ultrafast photodetectors and 2D planar LEDs, have been recently demonstrated. Such rapid advance in this field reflects great potential of 2D semiconductors for emerging optoelectronics. Nevertheless, the current research stays at the level of a proof of concept for various optoelectronic devices. Therefore, it is still required to thoroughly study the underlying operation principles of the newly proposed devices, enabling the realization of high-performance 2D optoelectronic devices superior to the traditional counterparts. Furthermore, in order to move toward practical applications, we should address several critical issues and challenges: (1) development of scalable strategies for large-area materials growth and high-quality fabrication of heterostructures; (2) the controllable engineering of the material properties such as carrier density and bandgap energy; (3) establishment of long-term environmental material stability and device reliability; and (4) search for innovative applications that cannot be realized by conventional semiconductor optoelectronics. Despite many scientific and technological issues at present, if these challenges can be resolved, 2D semiconductors and their vdW heterostructures would offer unprecedented opportunities for applications in next-generation optoelectronic devices with optical transparency, mechanical flexibility, and capability of non-epitaxial integration.

## Figures and Tables

**Table 1:** A variety of 2D materials with different electrical properties.

**Metal**	Graphene	Group V TMDCs (VX_2_, NbX_2_, TaX_2_)			TiS_2_, NiSe_2_, PdS_2_, PdSe_2_, PtS_2_, PtSe_2_	
**Semiconductor**	Group VI TMDCs (MoX_2_, WX_2_)		ReX_2_, HfX_2_, ZrX_2_, TcX_2_, TiSe_2_, TiTe_2_, InSe, In_2_Se_3_, GaSe, GaTe, PtTe_2_			Black phosphorous
**Insulator**	*h*-BN	Graphene oxide		2D oxides (Ti_0.87_O_2_, LaNb_2_O_7_, (Ca,Sr)_2_Nb_3_O_10_, CaLaNb_2_TiO_10_, La_2_Ti_2_NbO_10_, etc)		

X = Chalcogen(S or Se or Te).

**Table 2:** Comparison between the atomically thin *p*-*n* junction and the conventional bulk *p*-*n* junction.

Atomically thin *p*-*n* junction	Conventional bulk *p*-*n* junction
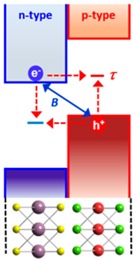	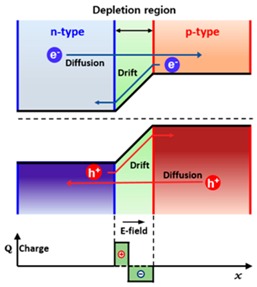
No extended depletion region Tunneling-mediated interlayer recombination Ultrafast change transfer (≤1 ps)	Depletion region (~a few hundred nm) Diffusion & drift of carriers

## Abbreviations

The following abbreviations are used in this manuscript:

2D	Two-dimensional
TMDCs	Transition metal dichalcogenides
FETs	Field-effect transistors
*h*-BN	Hexagonal boron nitride
vdW	van der Waals
PL	Photoluminescence
3D	Three-dimensional
STEM	Scanning transmission electron microscopy
CVD	Chemical vapor deposition
LEDs	Light-emitting diodes
PMMA	Polymethyl methacrylate
PDMS	Polydimethylsiloxane
PPC	Polypropylene carbonate
EQE	External quantum efficiency
EL	Electroluminescence
MQWs	Multiple quantum wells
